# Road to Genicular Artery Embolization: Importance of the Anastomotic Network

**DOI:** 10.1007/s00270-025-04121-8

**Published:** 2025-07-24

**Authors:** A. Taheri Amin, F. Ziayee, M. Boschheidgen, A. Hübner, E. Kemmer, D. Weiss, L. M. Wilms, E. Tietz, K. Jannusch, P. Minko

**Affiliations:** Department of Diagnostic and Interventional Radiology, Medical Faculty, University Duesseldorf, Moorenstrasse 5, 40225 Duesseldorf, Germany

**Keywords:** Genicular artery embolization, Anastomotic network, Anastomoses, Alternative access routes, Retrograde embolization, Antegrade embolization, Cross flow, R-GAE, A-GAE

## Abstract

**Purpose:**

To explore feasibility, safety, perfusion changes and clinical outcomes following retrograde genicular artery embolization (R-GAE).

**Materials and Methods:**

This prospective exploratory study assessed the technical approach, perfusion changes and clinical outcomes after R-GAE. Anastomoses between genicular arteries (GA) were evaluated during angiography. Technical success was defined as retrograde opacification of GA via anastomoses up to their origin, followed by embolization. Remaining GA were embolized antegrade (A-GAE), and additional A-GAE was performed after R-GAE (A-/R-GAE) if feasible. Time-density curves calculated peak intensity (PI), time-to-arrival (TTA), and area under the curve (AUC). Clinical outcomes were assessed using the knee injury and osteoarthritis outcome score (KOOS) at 6 weeks, 3 and 6 months. No comparison between R-GAE and A-GAE was performed.

**Results:**

A total of 132 vessels were embolized in 35 patients: 73 A-GAE, 39 R-GAE, and 20 A-/R-GAE. Technical success was achieved in all patients, with six cases of mild, transient skin discoloration. All KOOS subscales showed significant improvement at all time points (*p* < 0.05). No significant changes in PI, TTA, or AUC were observed in the parent vessels or anastomoses after GAE. Target vessels demonstrated reduced AUC (542.6 vs. 253.1; *p* < 0.01) and PI (110.3 vs. 51.2; *p* < 0.01), with increased TTA (7.1 s vs. 11.2 s; *p* < 0.01). Antegrade angiography after R-GAE revealed residual blush in all patients, requiring additional A-GAE.

**Conclusion:**

R-GAE is a safe and feasible catheterization route with potential efficacy at early follow-up. Perfusion areas reached by R-GAE may differ from A-GAE highlighting the anastomotic network’s impact on hemodynamics during GAE.

**Supplementary Information:**

The online version contains supplementary material available at 10.1007/s00270-025-04121-8.

## Introduction

Genicular artery embolization (GAE) is a novel treatment approach for knee osteoarthritis (OA) [1–4]. By targeting pathological neovascularization within the genicular arteries (GAs), GAE has demonstrated safety and efficacy in meta-analyses [5–9]. The primary GAs relevant to GAE include the descending GA (DGA), superomedial GA (SMGA), superolateral GA (SLGA), inferomedial GA (IMGA), inferolateral GA (ILGA) and anterior tibial recurrent artery (ATRA) [10, 11].

The technical aspects of GAE remain debated, particularly regarding which GAs should be catheterized. While some studies suggest targeting arteries based on primary pain localization [12], randomized controlled trials (RCTs) have reported that embolization of a single GA may trigger neovascularization in previously “normal” arteries [13].

This variability is reflected in clinical trials: the GAUCHO-Trial defines technical success as embolization of at least one GA [14], whereas the GENESIS-2-trial requires embolization of all GAs associated with pain/synovitis [15]. Landers et al. [13] and our previous study [16] adopted a more comprehensive approach, embolizing all visible GAs, underlining that the number of embolized GAs impacts the efficacy of GAE. A possible step toward answering which arteries should be targeted during GAE is a better understanding of the anastomotic network and its influence on hemodynamics.

Another technical challenge in GAE is the catheterization of certain arteries, such as the SMGA [10, 13]. Given its frequent anastomoses with the DGA, anastomotic pathways may offer a viable alternative catheterization route [10].

Angiographic [11, 17] and cadaveric studies [12, 18] have demonstrated the extensive anastomotic network surrounding the knee joint. However, to the best of our knowledge, anastomoses have only been described in the context of non-target embolization. Catheterization of anastomoses for retrograde GAE (R-GAE) has not been investigated yet. Superselective angiography of anastomoses provides direct insight into flow dynamics within these vessels. Thus, the aim of this study was to evaluate the feasibility and safety of R-GAE and to investigate anastomoses as an alternative access route during GAE.

## Materials and Methods

This prospective, single-center exploratory study was conducted at Duesseldorf University Hospital, enrolling knee OA patients undergoing GAE between January and December 2024. Inclusion criteria were: (1) age between 18 and 100 years, (2) GAE performed for refractory knee pain, and (3) at least one GA embolized retrograde via anastomoses. Cases with poor image quality inadequate for anatomical assessment were excluded. The study was approved by the Institutional Review Board and conducted in accordance with the Declaration of Helsinki.

### Interventional Procedure

Three interventional radiologists (Years of experience: P.M.: 18; F.Z.: 12, K.J.: 5) performed all interventions in an outpatient setting. Ipsilateral antegrade transfemoral access was obtained without the use of an introducer sheath to minimize the size of vascular access. Digital subtraction angiography (DSA) was performed at the mid-third of the distal superficial femoral artery (SFA) via a 4F-Cobra-catheter (Infiniti®, Cordis Medical, Austria) using iodinated contrast media (300 mg/mL Accupaque ®, GE HealthCare, USA) to visualize the vascular anatomy. Superselective catheterization of the GAs was performed using a 1.7F microcatheter (Pursue®, Merit Medical, USA). If superselective angiography revealed anastomoses to other GAs, retrograde catheterization was attempted. Technical success was defined as retrograde contrast filling of a GA via its anastomosis up to its origin from the popliteal artery (PA), SFA or anterior tibial artery (R-GAE). If a GA was successfully catheterized via its anastomosis, embolization was performed upon detection of a hyperemic blush using permanent embolic material (100–300 μm Embospheres®, Merit Medical, USA) diluted in 10 mL of the iodinated contrast agent mentioned above. During embolization, aliquots of the embolic mixture were injected into the target vessels (TVs) to “prune” abnormal neovessels while preserving normal inflow of the parenting vessel (PV) and anastomoses (AV). If no anastomoses were identified, embolization was performed only antegrade (A-GAE). In cases where vessels were embolized retrograde but had a prominent origin, they were additionally catheterized antegrade and re-embolized if necessary (R-/A-GAE). No randomization between A-GAE and R-GAE was conducted. A final full-view DSA series was performed at the mid-third of the distal SFA to ensure preservation of GA origins and assess for spontaneous opening of new vessels. To minimize non-target embolization, ice packs were applied around the knee joint during embolization. Patients were observed in the outpatient clinic for at least four hours before discharge.

Procedural parameters were recorded in a standardized report. Vascular and non-vascular complications were assessed upon discharge and 24 h post-procedure with duplex ultrasound and clinical evaluation. Complications were categorized according to the Cardiovascular and Interventional Radiological Society of Europe [19]. Clinical outcomes were assessed using the Knee injury and Osteoarthritis Outcome Score (KOOS) at 6 weeks, 3 and 6 months. Clinical failure was defined by a lack of improvement or deterioration in KOOS scores at any follow-up visit. No comparison between R-GAE and A-GAE was performed.

### Imaging Review and Data Analysis

**Fig. 1:**
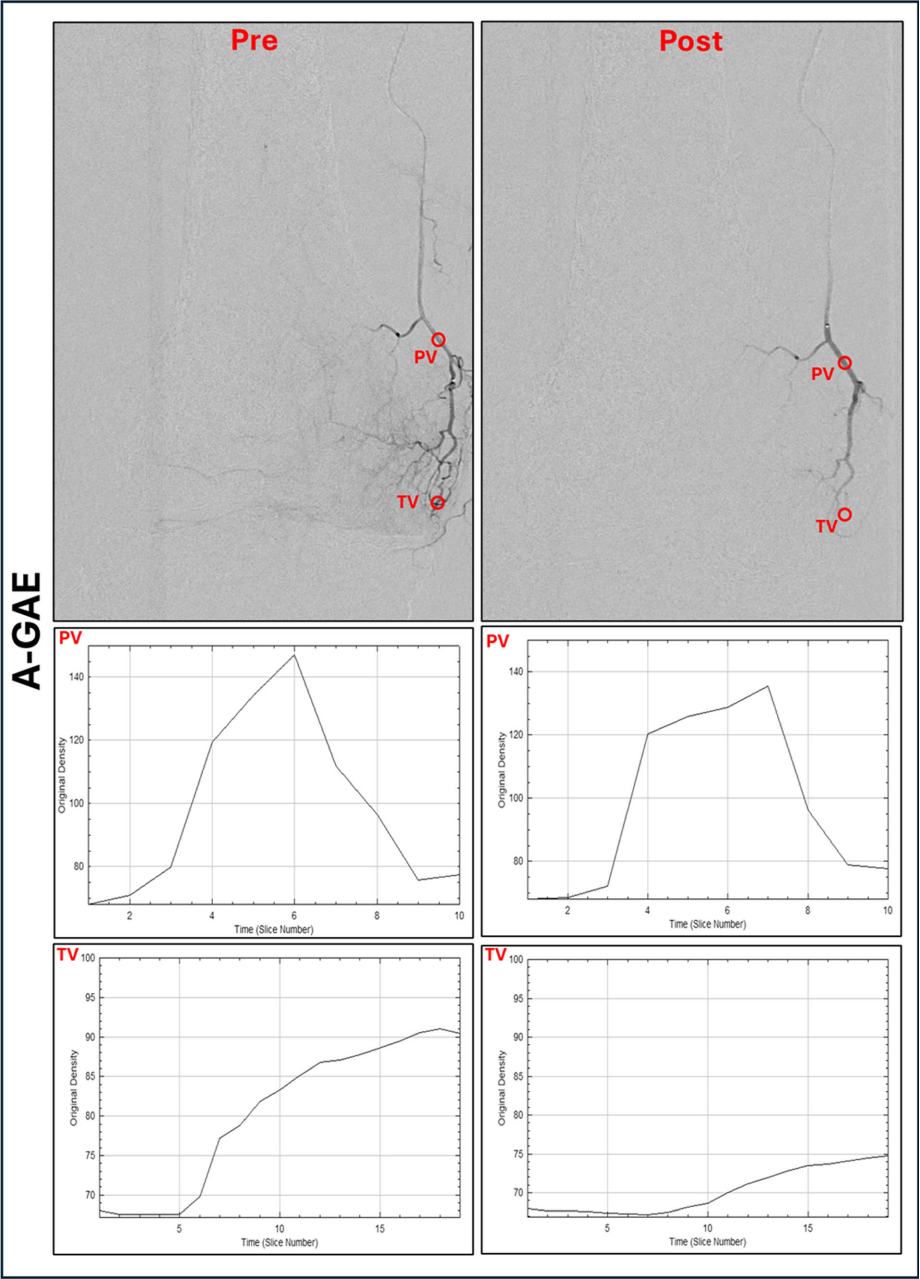

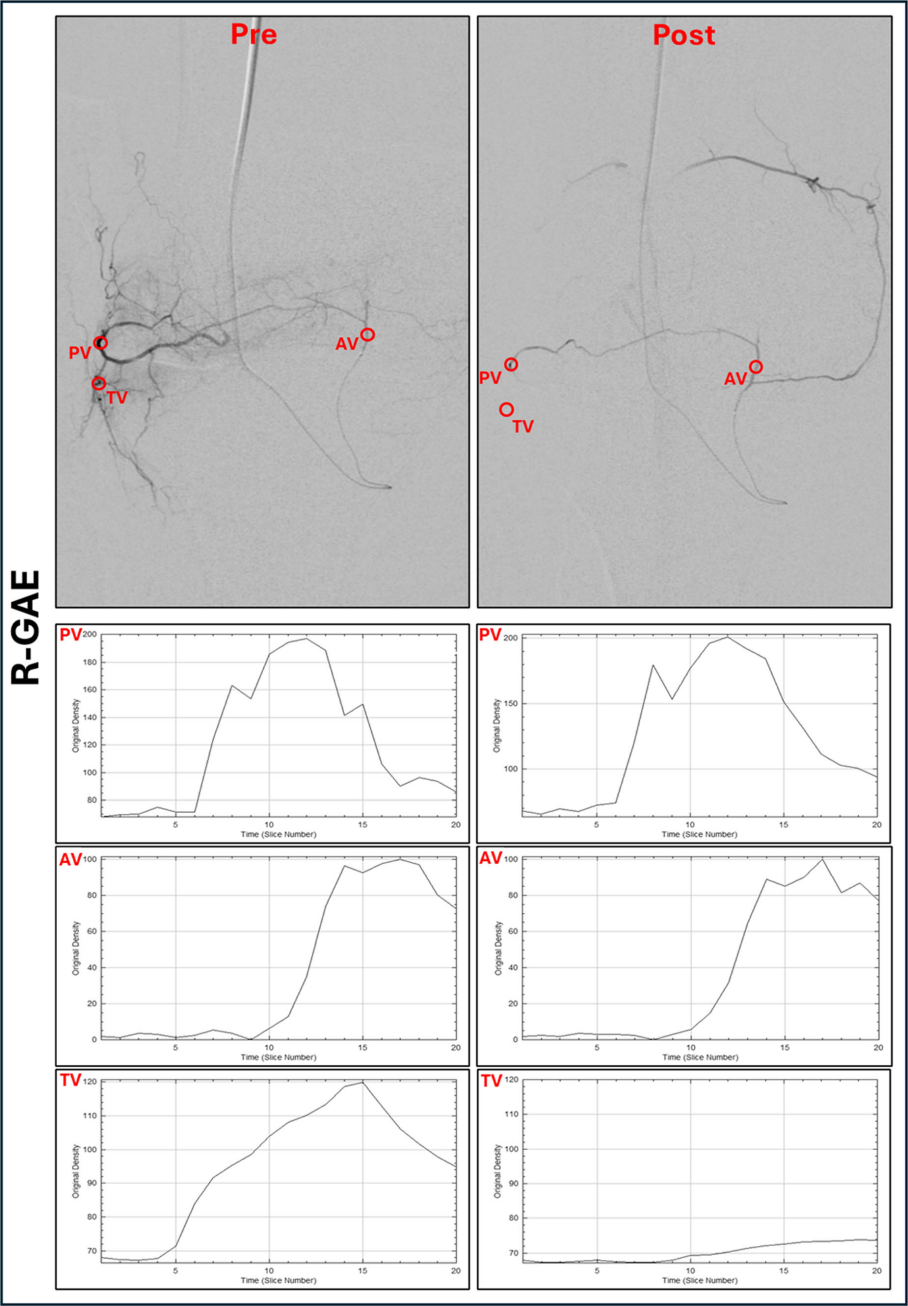
Quantitative perfusion parameters in ante- and retrograde GAE. Regions of interest (ROIs) were placed in the parenting vessel (PV) and target vessel (TV) before (pre) and after (post) antegrade embolization (A-GAE). In vessels embolized retrograde (R-GAE) ROIs were placed in the PV, TV and the anastomoses (AV) before and after embolization. Corresponding time-density curves were drawn and are shown below. **A** A-GAE of the descending genicular artery (DGA) with 1.5 mL of Microspheres (100–300 μm Embospheres, Merit Medical, USA). The time-density curves of the PV show no significant change after A-GAE, while those of the TV decrease. **B** R-GAE of the inferiorlateral genicular artery (ILGA) via the inferiomedial genicular artery (IMGA). After embolization with 1 mL of the above-mentioned microspheres, collaterals between the IMGA and the superiomedial genicular artery (SMGA) are seen. The time-density curves of the PV and AV show no significant change after R-GAE, while those of the TV decrease. ROI, Region of interest; PV, Parent vessel; AV, Anastomotic vessel; TV, Target vessel; A-GAE, Antegrade genicular artery embolization; R-GAE, Retrograde genicular artery embolization; DGA, Descending genicular artery; SMGA, Superiomedial genicular artery; IMGA: Inferiomedial genicular artery; ILGA, Inferiolateral genicular artery

Knee radiographs were reviewed by board-certified radiologists in consensus using the Kellgren-Lawrence scale (K&L). DSA images were reviewed by two radiologists (A.T., E.K.) assessing anastomoses between GAs. Images were transferred to ImageJ (National Institutes of Health, Bethesda, Maryland) for postprocessing. For each treated vessel, regions of interest (ROI) were defined, including the lumen of the feeding parent vessel (PV) supplying the hyperemic vessel(s), the hyperemic target area/vessel(s) (TV) and, in cases of R-GAE, within the retrograde catheterized vessel as PV, the adjacent hyperemic areas/vessel(s) as TV and in the anastomoses (AV). Identical circular ROIs, matching in shape, size, and location, were applied to both pre- and post-GAE angiograms, as shown in Fig. 1. Each ROI was placed by two radiologists (T.A., E.K.) and reviewed. Any discrepancies were to be resolved by a third radiologist (L.W.), though no such discrepancies were found.

Time-density curves were drawn for each ROI using the ImageJ plugin, “PaC DSA” as described by Zhao et al. [20].The following perfusion parameters were computed for each ROI before and after GAE using Excel 2412 (Microsoft, Washington, USA): area under the curve (AUC) for measuring total blood volume; peak intensity (PI) for measuring highest blood concentration; and time-to-arrival (TTA) for measuring blood velocity [21]. In addition to retrograde contrast filling of a GA, technical success required a post-embolization decrease in AUC and PI, along with an increase in TTA in the TV, while values in the PV remained unchanged. Perfusion parameters were thus used to confirm effective pruning of TVs while preserving PVs and AVs, serving as markers of technical feasibility.

In cases of R-/A-GAE, changes in TTA, PI, and AUC before and after embolization were calculated for both the PV and TV. Differences in TTA, PI, and AUC in PVs and TVs were then compared between A-GAE and R-GAE.

**Fig. 2:**
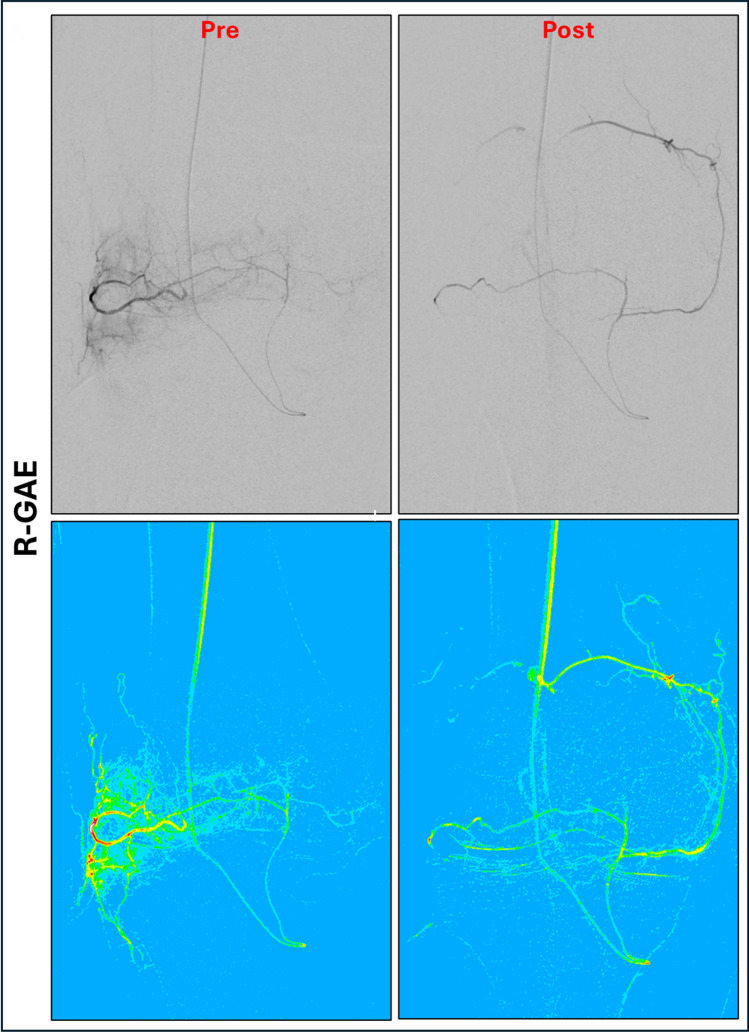

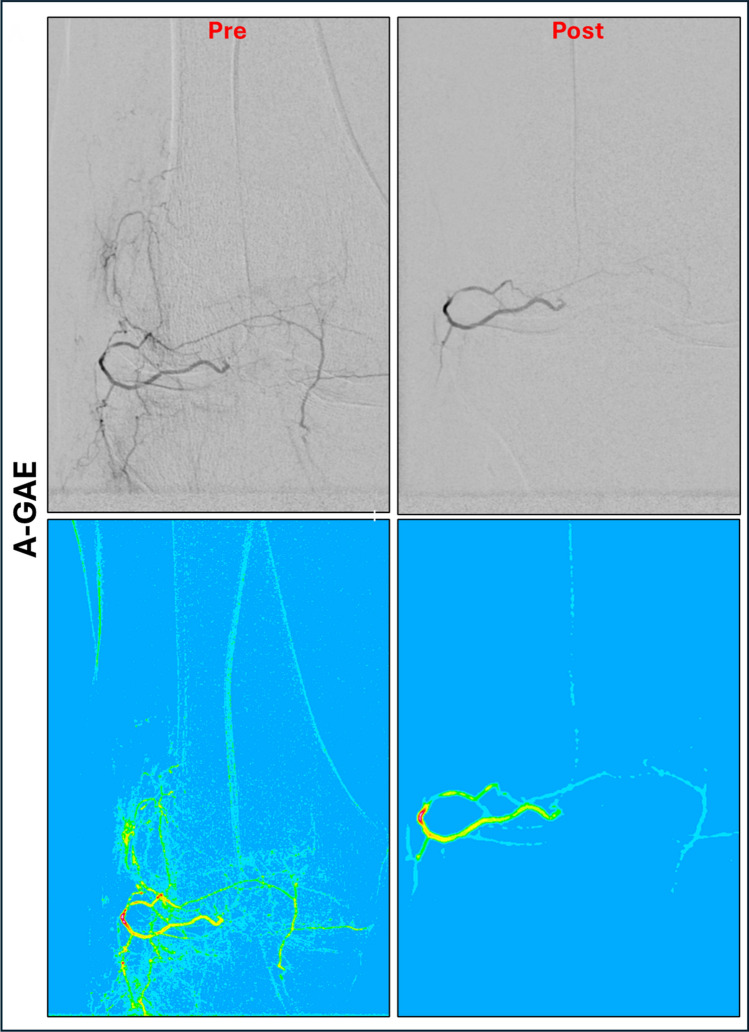
Color-coded DSA images of vessels embolized retro- and antegrade. DSA Images (above) and color-coded DSA images (below) of retro- and antegrade embolization of the inferiolateral genicular artery (ILGA). After retrograde embolization (R-GAE) of the ILGA via the inferiomedial genicular artery (IMGA) with 1.0 mL of Microspheres (100–300 μm Embospheres, Merit Medical, USA) adequate pruning of the neovessels with preserved flow of the ILGA and the anastomosis can be seen. Subsequent antegrade angiography (A-GAE) revealed a persistent blush, which was treated with an additional 1.5 mL of embolic. ILGA, Inferiolateral genicular artery; IMGA, Inferiomedial genicular artery; R-GAE, Retrograde genicular artery embolization; A-GAE, Antegrade genicular artery embolization

Furthermore, color-coded DSA images (ccDSA) were generated pre- and post-GAE using ImageJ [20], as seen in Fig. 2. The ccDSA images were anonymized and reviewed by two interventional radiologists (L.W.; D.W.), who qualitatively compared blush location and size during R-GAE with that observed during A-GAE. The following evaluation criteria were used: no blush, persistent blush and differing blush pattern.

Given the exploratory design focused on evaluating feasibility and safety, no formal sample size calculation was performed. Continuous variables were tested for normality using the Shapiro–Wilk test. For normally distributed data, paired t-tests were applied. If normality was not confirmed, the non-parametric Wilcoxon signed-rank test was used instead. Results were considered statistically significant at a two-sided *p*-value < 0.05. Statistical analyses were performed using Prism 10.4.2 (GraphPad Software, San Diego, USA).

## Results

A total of 35 patients suffering from OA Grade II to IV and post-TKA pain were included in the study. Patient characteristics are shown in Table 1.

**Table 1:** Patient characteristics

Number of patients (n)	35
Age (years) median (range)	62 (37–85)
Female, n (%)	21 (60%)
BMI, median (range)	26 (20–40)
Kellgren–Lawrence grade (n)	
I	0
Il	11
III	13
IV	9
TKA	2

BMI, Body mass index; TKR, Total knee replacement

Anastomoses between GAs were seen in all patients. Table 2 summarizes the frequency and mean diameter of anastomoses within the medial and lateral compartments as well as between compartments. No differences in anastomotic diameters between compartments were observed.

**Table 2:** Anatomic analysis of anastomoses

	Frequency n (%)	Diameter(mean ± SD; cm)
Medial		
DGA + SMGA	7 (20%)	0.4 ± 0.1
DGA + IMGA	11 (31%)	0.5 ± 0.1
SMGA + IMGA	6 (17%)	0.4 ± 0.2
Lateral		
SLGA + ILGA	8 (23%)	0.4 ± 0.1
SLGA + ARTA	1 (3%)	0.5 ± 0.1
ILGA + ARTA	1 (3%)	0.4 ± 0.1
Medial/lateral		
DGA + SLGA	3 (9%)	0.4 ± 0.1
DGA + ILGA	8 (23%)	0.5 ± 0.1
SMGA + ILGA	2 (6%)	0.3 ± 0.1
IMGA + ILGA	12 (34%)	0.5 ± 0.1

DGA, Descending genicular artery; SMGA, Superiomedial genicular artery; IMGA, Inferiomedial genicular artery; SLGA, Superiolateral genicular artery; ILGA, Inferiolateral genicular artery; ARTA, Anterior recurrent tibial artery

**Fig. 3:**
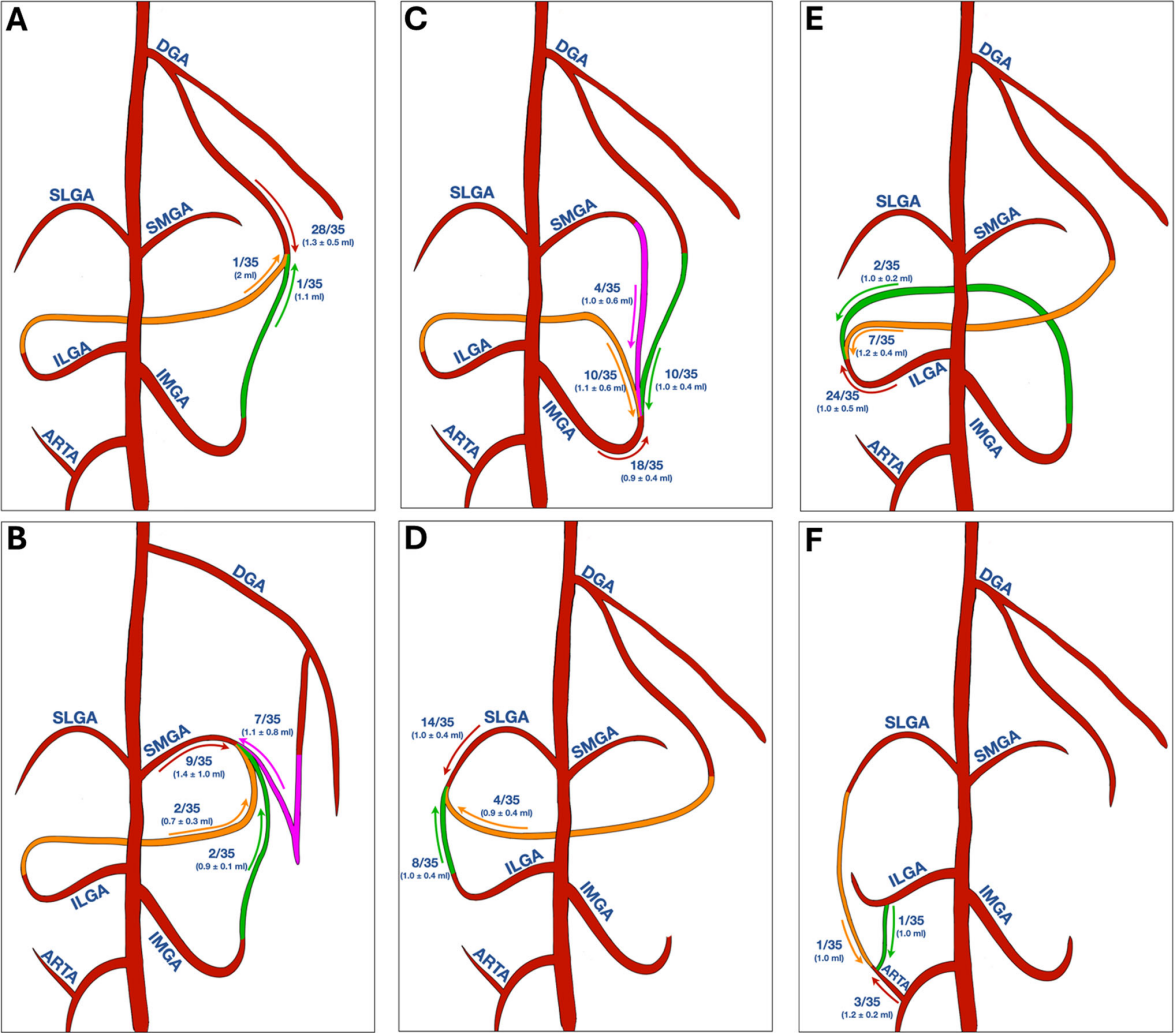
Frequency and volume of antegrade and retrograde genicular artery embolization. For each genicular artery, the frequency (top row) and embolic volume (bottom row; mean ± SD) are shown separately for antegrade embolization (A-GAE; red) and retrograde embolization (R-GAE; green, orange, and purple). **A** R-GAE of the descending genicular artery (DGA) was performed via the inferiolateral genicular artery (ILGA) and via the inferiomedial genicular artery (IMGA). **B** R-GAE of the superomedial genicular artery (SMGA) was performed via the DGA, IMGA and ILGA. **C** R-GAE of the IMGA was performed via the DGA, SMGA and ILGA. **D** R-GAE of the superiolateral genicular artery (SLGA) was performed via the DGA and ILGA. **E** R-GAE of ILGA was performed via the DGA and IMGA. **F** R-GAE of the anterior recurrent tibial artery (ARTA) was performed via the SLGA and ILGA. A-GAE, Antegrade genicular artery embolization; R-GAE, Retrograde genicular artery embolization; DGA, Descending genicular artery; SMGA, Superiomedial genicular artery; IMGA, Inferiomedial genicular artery; SLGA, Superiolateral genicular artery; ILGA, Inferiolateral genicular artery; ARTA, Anterior recurrent tibial artery

In total, 132 vessels were embolized with 73 vessels (55%) using A-GAE, 39 (30%) R-GAE and 20 (25%) R-/A-GAE. In all R-GAEs, technical success was achieved. The median number of embolized vessel per patient was four (range: 2–6), with a median of two vessels (11–4 vessels) in A-GAE, one vessel (0–3 vessels) in R-GAE and one vessel (0–2 vessels) in A-/R-GAE. The mean total embolic volume administered per patient was 4.7 ± 1.1 mL (2.1–6.9 mL), with 3.3 ± 1.4 mL (0.9–5.9 mL) in A-GAE and 1.7 ± 1.2 mL (0.4–5.9 mL) in R-GAE. Figure 3 illustrates the frequency and embolic volume of A-GAE and R-GAE for each GA, including the retrograde access used.

Mean cumulative air kerma was 45 ± 26 mGy (14–108 mGy) and mean fluoroscopy time 19 ± 8 min (9–38 min).

Due to non-normal distribution, perfusion parameters, were assessed using the Wilcoxon signed-rank test.

**Fig. 4:**
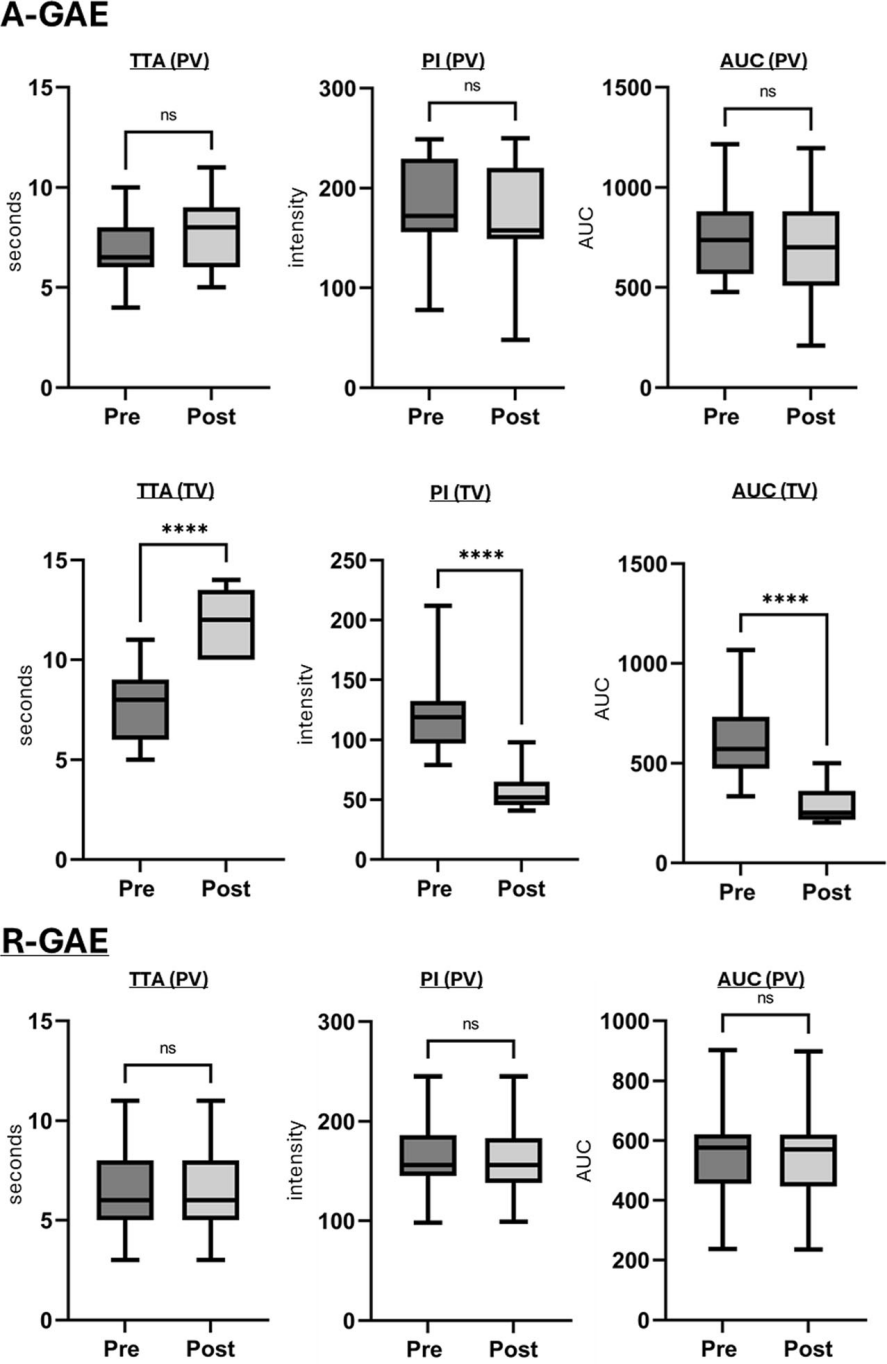

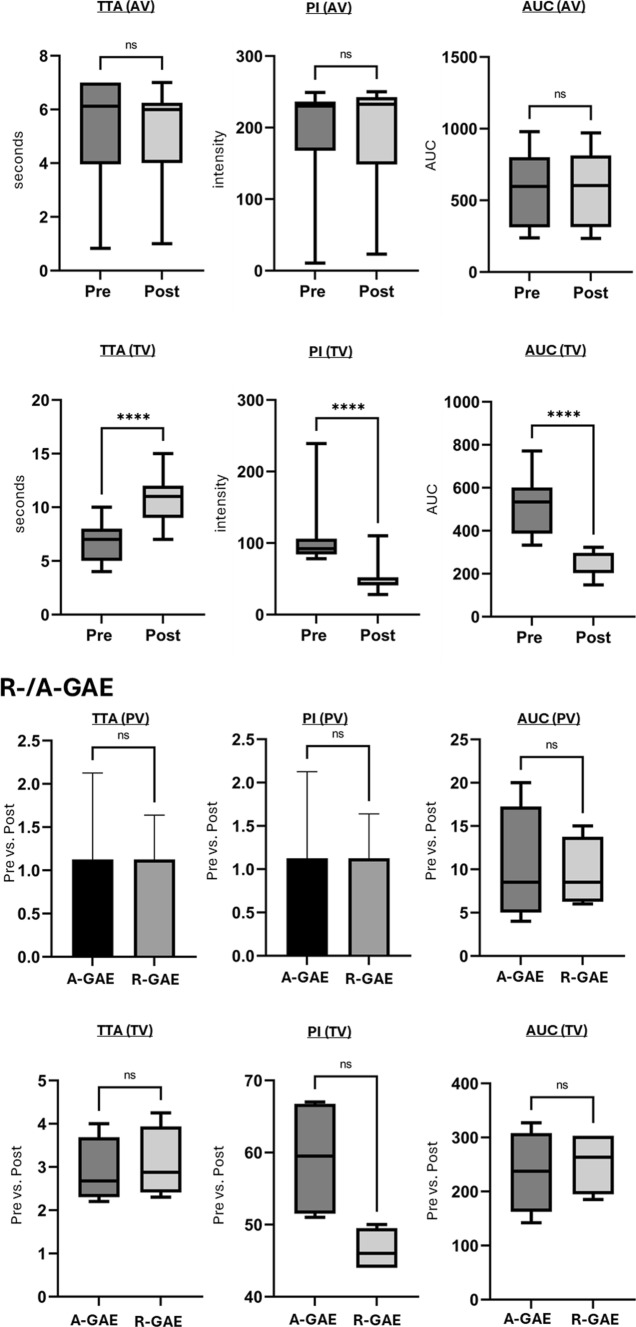
Quantitative perfusion parameters in ante- and retrograde genicular artery embolization.** A** In antegrade GAE (A-GAE) no significant (ns) changes in time-to-arrival (TTA), peak intensity (PI) and area under the curve (AUC) were observed in the parenting vessel (PV). Target vessels (TV) showed significant (****: *p* ≤ 0.0001) increase in TTA and decrease in PI and AUC. **B** In retrograde GAE (R-GAE) no significant changes in PI, TTA, or AUC were observed in the PV as well as in the anastomotic vessel (AV). TVs showed significant (*p* ≤ 0.0001) increase in TTA and decrease in PI and AUC. **C** In vessels embolized both retro- and antegrade (R-/A-GAE) changes of TTA, PI and AUC in the PV after embolization showed no significant differences between A- and R-GAE. In the TVs increase in TTA and decrease in PI and AUC showed no significant differences between A-GAE and R-GAE. TTA, Time-to-arrival; PI, Peak intensity; AUC, Area under the curve; PV, Parent vessel; AV, Anastomotic vessel; TV, Target vessel; A-GAE, Antegrade genicular artery embolization; R-GAE, Retrograde genicular artery embolization; R-/A-GAE, Retrograde and antegrade genicular artery embolization

In A-GAE (Fig. 4A; Table 3), mean TTA, PI and AUC in the PV showed no significant changes after GAE. In the TV, mean TTA increased significantly (*p* < 0.001), while PI and AUC decreased significantly (*p* < 0.0001) after A-GAE.

**Table 3:** Quantitative perfusion parameters in A- and R-GAE

	Pre-embolization			Post-embolization		
	TTA(mean ± SD)	PI(mean ± SD)	AUC(mean ± SD)	TTA(mean ± SD)	PI(mean ± SD)	AUC(mean ± SD)
A-GAE						
PV	6.9 ± 1.6 s	182 ± 47	767 ± 231	7.3 ± 2.1 s	171 ± 55	717 ± 276
TV	7.8 ± 1.6 s	122.0 ± 32.4	609.0 ± 181.2	12.0 ± 1.5 s	57.2 ± 16.4	294.2 ± 86.7
R-GAE						
PV	6.6 ± 2.1 s	164.2 ± 42.1	536.2 ± 167.1	6.8 ± 1.8 s	163.1 ± 41.8	531.2 ± 168.2
AV	6.9 ± 1.3 s	173.1 ± 32.3	423.1 ± 123.3	7.1 ± 1.1 s	153.7 ± 28.3	403.1 ± 134.1
TV	6.8 ± 1.8 s	105 ± 39.3	513.1 ± 129.3	10.8 ± 2.1 s	49.4 ± 18.8	231.1 ± 57.1
R-GAE & A-GAE						
PV	6.3 ± 0,8 s	232.2 ± 10.5	650.2 ± 238.3	6.3 ± 0.8 s	229 ± 11.2	656 ± 235.3
TV	8.9 ± 1.3 s	104.2 ± 10.6	418.2 ± 109.1	12.3 ± 1.1 s	44.8 ± 4.3	182.2 ± 48.5
PV	6.3 ± 1.1 s	221.2 ± 11.6	631.1 ± 228.3	6.3 ± 1.1 s	219.2 ± 12.3	628.2 ± 226.3
TV	8.3 ± 2.2 s	93.3 ± 9.9	444.3 ± 91.2	12.1 ± 2.4 s	46.8 ± 8.8	190.3 ± 40.8

Time-to-arrival (TTA), peak intensity (PI), and area under the curve (AUC) showed no significant changes after embolization in the parent vessels (PV) and the anastomoses (AV) catheterized antegrade (A-GAE), retrograde (R-GAE) or both (R-/A-GAE). In the target vessels (TV), TTA significantly increased, while PI and AUC significantly decreased following A-GAE, R-GAE and R-/A-GAE. TTA, Time-to-arrival; PI, Peak intensity; AUC, Area under the curve; PV, Parent vessel; AV, Anastomotic vessel; TV, Target vessel; A-GAE, Antegrade genicular artery embolization; R-GAE, Retrograde genicular artery embolization; R-/A-GAE, Retrograde and antegrade genicular artery embolization

In R-GAE (Fig. 4B; Table 3), mean TTA, PI and AUC in the PV and AV showed no significant changes after GAE. In the TV, mean TTA increased significantly (*p* < 0.0001), while mean PI and AUC decreased significantly (*p* < 0.0001) following R-GAE.

In R-/A-GAE (Fig. 4C; Table 3), mean TTA, PI and AUC of the PV remained unchanged after GAE, with no significant differences observed between R-GAE and A-GAE. In the TV, mean TTA increased significantly after R-/A-GAE (*p* < 0.0001), with no significant differences between R-GAE and A-GAE. Similarly, PI and AUC decreased significantly (*p* < 0.0001) in the TV, with no significant differences between the two techniques.

The ccDSA images revealed differences in blush location (*n* = 5) or a persistent blush (*n* = 15) following R-/A-GAE.

**Fig. 5:**
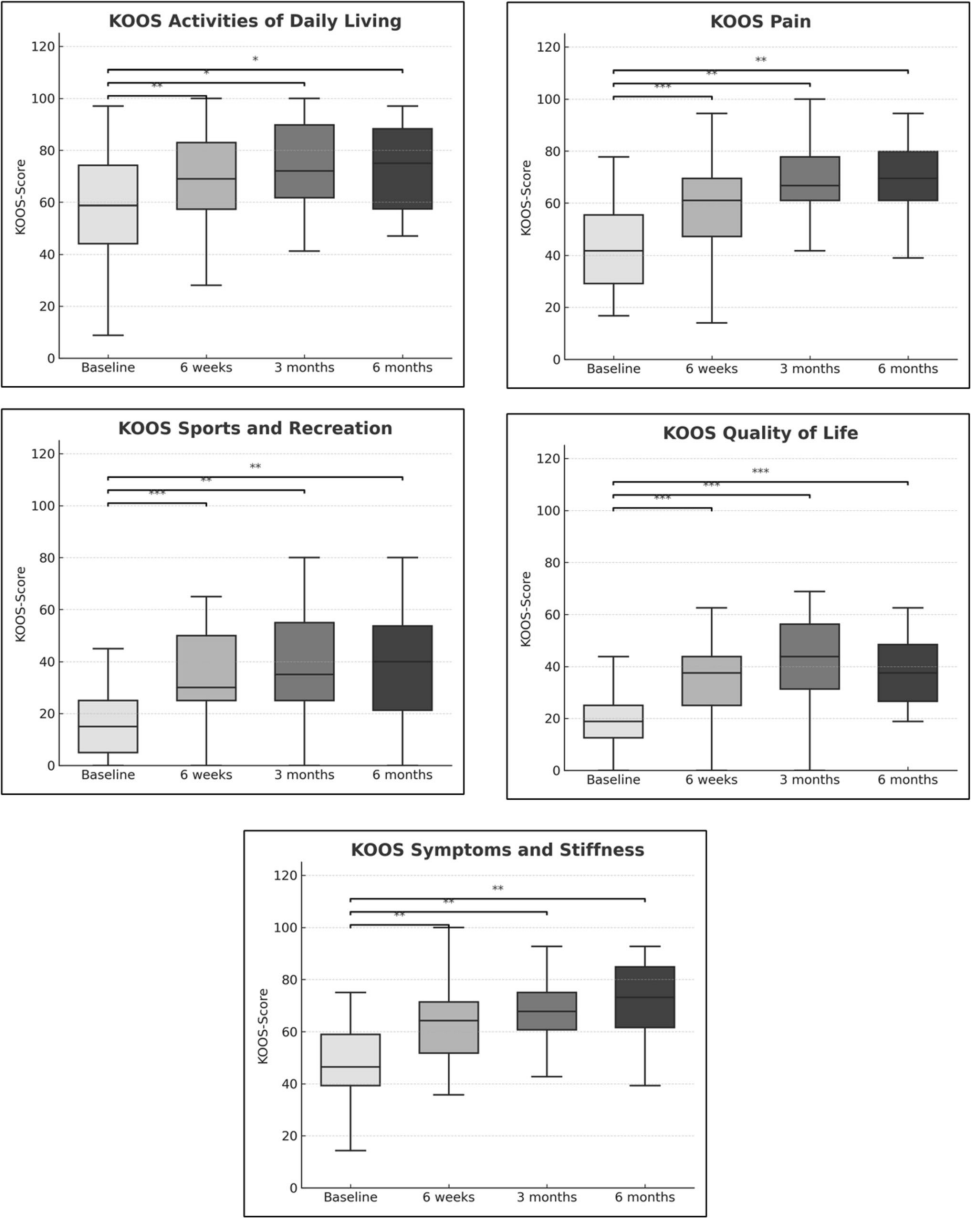
Boxplots of Knee Injury and Osteoarthritis Outcome Scores (KOOS) subscales. Boxplots depicting: KOOS subscale daily living, KOOS subscale sports and recreation, KOOS subscale pain, KOOS subscale quality of life and KOOS subscale symptoms and stiffness. (*: p ≤ 0.05; **: p ≤ 0.01; ***: p ≤ *0.001).* KOOS, Knee Injury and Osteoarthritis Outcome Score

All patients completed clinical follow-up at every time point. Since KOOS scores were normally distributed, comparisons between time points were performed using the paired t-test. All KOOS subscales demonstrated statistically significant improvement from baseline at 6 weeks, 3 months and 6 months (Fig. 5; Table 4). In all patients, the origins of all GAs were preserved during the final DSA series.

**Table 4:** Mean knee injury and osteoarthritis outcome scores (KOOS) subscales

Subscale	Visit	Mean score ± SD	*p*-value
Daily living	Baseline	57.4 ± 23.0	
	6 weeks	67.6 ± 21.0	< 0.01
	3 months	72.2 ± 20.7	< 0.05
	6 months	73.3 ± 17.3	< 0.05
Sports and recreational activities	Baseline	17.6 ± 14.7	
	6 weeks	37.0 ± 23.3	< 0.001
	3 months	38.3 ± 22.6	< 0.01
	6 months	39.6 ± 23.8	< 0.01
Pain	Baseline	43.7 ± 17.6	
	6 weeks	58.1 ± 17.5	< 0.001
	3 months	65.7 ± 19.4	< 0.01
	6 months	68.4 ± 15.1	< 0.01
Quality of life	Baseline	19.9 ± 15.3	
	6 weeks	35.2 ± 17.1	< 0.001
	3 months	42.6 ± 18.4	< 0.001
	6 months	38.8 ± 13.2	< 0.001
Symptoms and stiffness	Baseline	47.9 ± 16.8	
	6 weeks	62.3 ± 18.3	< 0.01
	3 months	66.0 ± 16.4	< 0.01
	6 months	71.1 ± 17.3	< 0.01

Mild skin discolorations occurred in six patients and resolved completely by six weeks. In all cases one GA was embolized via R-GAE, while others were treated via A-GAE. One patient received combined R-/A-GAE. No association was found between the frequency of skin discolorations and embolization technique.

## Discussion

This study provides evidence that R-GAE is a safe and feasible approach, with efficacy at early follow-up.

Consistent with previous studies [10, 12, 22], the most frequently observed and retrograde embolized vessels in our study were the IMGA and ILGA via the DGA, the SMGA via DGA and the SLGA via ILGA.

Among the GAs, the SMGA is particularly challenging to catheterize due to its small caliber and tortuous course [10, 13, 16], often requiring lateral projections and still failing in some cases [11]. In our study, the median number of embolized GAs was four—higher than in other trials [2, 23]—yet radiation dose and fluoroscopy time remained comparable. This may be explained by the more frequent retrograde catheterization of the SMGA and IMGA, suggesting R-GAE is a safe alternative when antegrade access is challenging.

Following both A-GAE and R-GAE, perfusion parameters in the PV remained unchanged. In the TV, TTA increased while PI and AUC decreased. The changes align with findings by Badar et al. [24] and were further validated in our larger cohort. In vessels treated with R-/A-GAE, no significant differences in perfusion parameter changes in the TV and PV were observed between R-GAE and A-GAE. This suggests that, from a technical standpoint, embolization endpoints appear equally achievable via R-GAE and A-GAE.

In all R-/A-GAEs, subsequent antegrade angiography revealed persistent blush, requiring repeated A-GAE despite prior R-GAE. This suggests that the perfusion territories reached via R-GAE may differ from those of A-GAE. One possible explanation is the extensive anastomotic network of the knee, which gives rise to a broad collateral circulation [10–12]. While this may increase safety by reducing non-target embolization, it may also limit efficacy by maintaining hyperperfusion in a treated compartment through collateral flow from an untreated compartment. If a vascular territory can be embolized retrogradely via another, it is likely that it can also be reperfused in the same manner. Landers et al. observed that embolization of a single dominant GA led to spontaneous opening of additional neovessels in previously unaffected other GAs [13], prompting a protocol change to treat all GAs with blush. In the final analysis, only this group showed significant improvement over placebo, suggesting that the number of embolized arteries influences clinical outcome. Our data provides an angiographic correlate to this observation that may explain the lack of clinical success after single-vessel GAE. Given the high degree of collateralization, it may be worth reconsidering the compartment-based approach to GAE. Rather than targeting individual GAs based on clinical symptoms, future strategies could explore evaluating the entire genicular network angiographically and embolizing vessels as needed.

However, this interpretation is limited by the fact that R-GAE was performed immediately after successful retrograde catheterization, while A-GAE was only added if easily feasible. As a result, antegrade angiography was conducted after R-GAE, limiting the comparability of blush patterns between techniques.

Other limitations include the use of single-plane angiographic projections, which may have underestimated the true extent of vascular blush. Manual contrast injection introduced variability in injection pressure and volume, limiting interindividual comparability of perfusion parameters. Subgroup analyses based on anatomical variations were not feasible due to the limited sample size and the heterogeneity of genicular anastomoses.

Although clinical outcomes following A- and R-GAE were reported, no direct comparison was made with patients treated exclusively with A-GAE. This represents a major limitation, as it reduces the ability to draw definitive conclusions about the specific clinical value of R-GAE. This remains an objective of further studies. The qualitative assessment of blush patterns is also subject to potential observer bias, despite efforts to blind raters to patient identity. Similarly, while all procedures were technically successful, the depth and consistency of catheterization may have varied depending on operator experience.

This study demonstrates technical feasibility and safety of R-GAE and its potential efficacy in combination with A-GAE at early follow-up. Anastomoses may serve as alternative retrograde access routes when antegrade catheterization is challenging.

Persistent blush after R-GAE requiring repeated A-GAE highlights the impact of the anastomotic network on hemodynamics and collateral flow during GAE. Thus, embolizing all visible genicular arteries may be necessary to reduce collateral inflow and achieve sustained devascularization of the knee joint.

## Supplementary Information

Below is the link to the electronic supplementary material.

Supplementary file1 (DOCX 26 KB)
