# Supplementary material for: Road to Genicular Artery Embolization: Importance of the Anastomotic Network
Source: Cardiovasc Intervent Radiol. 2025 Jul 24;48(12):1801–15. doi: 10.1007/s00270-025-04121-8 (PMC12665632; doi:10.1007/s00270-025-04121-8)
Supplement: Supplementary file 1 — Supplementary file1 (DOCX 26 KB) [file 270_2025_4121_MOESM1_ESM.docx]

**Supplements:**

**Table 1: Intervention data**

Genicular arteries were embolized using Microspheres (100-300 μm Embospheres, Merit Medical, USA) diluted in 10 mL of iodinated contrast agent (300mg/mL Accupaque, GE HealthCare, USA ). Embolic volume (in mL) injected antegrade (A-GAE) and retrograde (R-GAE) in each genicular artery per patient are listed below

Abbreviations DGA: Descending genicular artery; SMGA: Superiomedial genicular artery; IMGA: Inferiomedial genicular artery; SLGA: Superiolateral genicular artery; ILGA: Inferiolateral genicular artery; ARTA: Anterior recurrent tibial artery.

|  | **DGA** | | **SMGA** | | **IMGA** | | **SLGA** | | **ILGA** | | **ARTA** | |
| --- | --- | --- | --- | --- | --- | --- | --- | --- | --- | --- | --- | --- |
|  | A-GAE | R-GAE | A-GAE | R-GAE | A-GAE | R-GAE | A-GAE | R-GAE | A-GAE | R-GAE | A-GAE | R-GAE |
| 1 | 1.3 |  |  | 0.3 |  | 0.9 | 1.7 | 1.7 | 0.3 |  |  |  |
| 2 | 1 |  |  |  |  |  | 1.1 |  |  |  |  | 1 |
| 3 | 2 |  |  |  | 1.1 |  |  |  | 0.8 | 1.5 |  |  |
| 4 |  |  | 0.5 |  |  | 1.1 | 0.6 |  | 0.9 | 0.8 |  |  |
| 5 | 0.4 |  |  |  |  | 0.8 |  | 0.8 | 1.5 |  |  |  |
| 6 | 0.3 |  |  | 0.5 | 0.8 |  | 0.5 |  |  |  |  |  |
| 7 | 1 |  |  |  |  | 2 |  |  | 1.5 |  |  |  |
| 8 |  |  |  | 0.5 | 0.7 |  | 1 |  | 0.9 | 1.5 |  |  |
| 9 | 2.1 |  |  |  |  | 0.9 |  | 1.1 | 0.6 |  |  |  |
| 10 | 1.5 |  |  |  | 0.5 | 0.5 |  | 1.5 | 0.5 |  |  |  |
| 11 |  |  | 2 | 1 | 1 |  | 0.6 |  |  | 1 |  |  |
| 12 | 1 |  |  |  | 1 | 1 |  |  | 0.7 |  |  |  |
| 13 | 1.3 |  |  |  |  | 0.8 | 1 |  | 0.9 |  |  |  |
| 14 | 1.1 |  |  |  |  | 1.1 |  | 1.1 |  | 1.6 |  |  |
| 15 |  |  |  |  | 1.3 | 1 |  |  | 1 |  |  |  |
| 16 | 2.3 |  |  |  |  | 1.1 | 0.6 |  |  | 0.7 |  |  |
| 17 | 0.7 |  | 0.6 |  |  | 0.6 |  | 0.8 | 0.8 |  |  |  |
| 18 | 2.1 |  |  | 0.8 | 0.5 | 0.8 |  | 0.8 | 0.8 |  |  |  |
| 19 | 0.7 |  |  |  | 0.7 | 1.2 |  |  | 0.4 |  |  |  |
| 20 |  |  |  | 2.8 |  | 2.6 |  | 0.5 | 1 |  |  |  |
| 21 | 2 |  |  |  | 2 | 0.4 |  |  | 1.5 |  |  |  |
| 22 | 1.9 |  |  | 1.8 | 0.5 |  |  |  |  |  |  |  |
| 23 | 1.9 |  |  | 0.4 |  |  | 1.4 |  | 0.5 |  |  |  |
| 24 | 1.5 |  |  |  |  | 0.3 | 1.5 |  |  | 1.5 |  |  |
| 25 | 0.9 | 2 |  | 0.9 |  | 1.2 |  |  |  |  |  |  |
| 26 | 0.8 |  |  |  | 1.5 |  | 1.7 |  | 1.5 | 0.8 |  |  |
| 27 | 1,5 |  | 3.9 |  | 1.1 |  |  | 1.2 |  |  |  |  |
| 28 | 0.9 |  |  | 0.8 |  | 0.8 | 1 |  | 1.1 |  |  |  |
| 29 | 1.4 | 1.1 |  |  |  | 1.5 |  | 0,7 |  | 1.1 |  |  |
| 30 |  |  | 1.1 |  |  | 0,7 |  | 1.2 | 2.3 |  |  |  |
| 31 |  |  | 0.7 |  | 0.5 | 0.7 | 0.9 |  | 1.3 |  |  |  |
| 32 | 1 |  | 1.4 | 1 | 1.4 |  |  |  |  |  | 1.5 |  |
| 33 | 1 |  | 1.5 |  | 0.3 | 0.4 | 1 | 0.3 | 1 |  | 1.1 |  |
| 34 | 1.1 |  | 1 |  | 0.6 |  |  |  | 1 |  | 1 | 1 |
| 35 | 1.5 |  |  |  | 0.5 | 1.9 |  |  | 1.5 |  |  |  |
